# Effect of exercise intensity and apnea on splenic contraction and hemoglobin increase in well-trained cross-country skiers

**DOI:** 10.1007/s00421-024-05428-z

**Published:** 2024-02-23

**Authors:** Hampus Lindblom, Frank Pernett, Erika Schagatay, Pontus Holmström

**Affiliations:** 1 Swedish Winter Sports Research Centre, Department of Health Sciences, Mid Sweden University, Östersund, Sweden; 2 Environmental Physiology Group, Department of Health Sciences, Mid Sweden University, Östersund, Sweden

**Keywords:** Spleen, Cross-country skiing, Endurance exercise, Oxygen-carrying capacity, Sports performance

## Abstract

The human spleen acts as a reservoir for red blood cells, which is mobilized into the systemic circulation during various conditions such as hypoxia and physical exertion. Cross-country (XC) skiers, renowned for their exceptional aerobic capacity, are regularly exposed to high-intensity exercise and local oxygen deficits. We investigated a putative dose-dependent relationship between splenic contraction and concomitant hemoglobin concentration ([Hb]) elevation across four exercise intensities in well-trained XC skiers. Fourteen male XC skiers voluntarily participated in a 2-day protocol, encompassing a serial apnea test and a $\dot{V}$O_2max_ test (day 1), followed by three submaximal exercise intensities on a roller skiing treadmill corresponding to 55, 70, and 85% of $\dot{V}$O_2max_ (day 2). Spleen volume was measured via ultrasonic imaging, and venous blood samples were used to determine [Hb] levels. Baseline spleen volume was similar (266(35) mL) for all conditions (NS). Notably, all conditions induced significant splenic contractions and transient [Hb] elevations. The $\dot{V}$O_2max_ test exhibited the most pronounced splenic contraction (35.8%, *p* < 0.001) and a [Hb] increase of 8.1%, while the 85% exercise intensity led to 27.1% contraction and the greatest [Hb] increase (8.3%, < 0.001) compared to baseline. The apnea test induced relatively smaller responses (splenic contraction: 20.4%, [Hb] = 3.3%, *p* < 0.001), akin to the response observed at the 70% exercise intensity (splenic contraction = 23%, [Hb] = 6.4%, *p* < 0,001) and 55% (splenic contraction = 20.0%, [Hb] = 4.8%, *p* < 0.001). This study shows a discernible dose-dependent relationship between splenic contraction and [Hb] increase with levels of exercise, effectively distinguishing between submaximal and maximal exercise intensity.

## Introduction

Previous research has shown that several mammals use their spleens as a reservoir for red blood cells (RBCs), facilitating hemoglobin concentration ([Hb]) regulation during situations of physiological stress. Barcroft and Stephens (1927) reported that dogs halved their spleen size during exercise, whereby the blood that was introduced into circulation stimulated by the contraction measured 20% of the total blood volume. Since then, additional animal studies have observed similar functions in racehorses (Persson et al. 1973), sheep, and Weddell seals (Qvist et al. 1986; Hurford et al. 1996). The same effect has been demonstrated in human divers (Hurford 1991; Schagatay et al 2001), and studies demonstrated an apnea-prolonging effect in individuals with spleens, which was not present in splenectomized individuals (Schagatay et al. 2001; Bakovic et al. 2003). However, in the human spleen, the percentage of total body RBC is much smaller compared with many animal species, with a maximum about 10%, giving rise to debate regarding the practical significance of the splenic contraction (Stewart and McKenzie 2002; Shephard 2016). Nonetheless, numerous studies have investigated the initiation and function of the human spleen contraction in a variety of stressful situations including: static and dynamic apnea (Bakovic et al. 2003; Schagatay et al. 2005), normobaric hypoxia (Richardson et al. 2008; Lodin-Sundström et al. 2021; Pernett et al. 2021), and hypobaric hypoxia (Holmström et al. 2020a, 2020b), during exercise in patients with chronic obstructive pulmonary disease (Schagatay et al. 2015) and exercise in healthy individuals (Laub et al. 1993; Holmström et al. 2021), showing that the human spleen is involved in situations and environments leading to oxygen deficiencies.

While exercise has been confirmed to result in splenic contractions in several studies (Froelich et al. 1988; Flamm et al. 1990; Laub et al. 1993; Stewart et al. 2003; Holmström et al. 2021), the magnitude of the splenic contraction resulting from various modes of exercise and the effect on human endurance performance are still unclear. Sperlich et al. (2015) found no enhancement in cycling performance following apnea-induced splenic contraction, while Bouten et al. (2019) reported accelerated oxygen uptake ($\mathop {\text{V}}\limits^{.}$O_2_) kinetics during short-duration cycling performance, but they did not observe any positive effects on performance. In addition, Jahic et al. (2019) tested different levels of exercise intensities on elite long-distance runners and reported the greatest splenic contraction occurring at a workload above the anaerobic threshold.

Cross-country (XC) skiing has been shown to be one of the most demanding aerobic sports with maximal oxygen uptake ($\mathop {\text{V}}\limits^{.}$O_2max_) > 80/70 (ml min^−1^ kg^−1^) for both elite-level men and women (Ingjer 1991; Tønnessen et al. 2015). In addition, Andersson et al. (2017) and Losnegard et al. (2012) emphasize that both gross efficiency (GE) and anaerobic capacity are primary key performance factors, indicating the diversity of the required physiological characteristics of XC skiing performance. Given that oxygen-carrying capacity represents a pivotal limiting factor of $\dot{V}$O_2max_ at sea level, as established in previous studies (Ekblom et al. 1972; Bassett and Howley 2000), and considering the positive correlation between [Hb] and $\dot{V}$O_2max_ as well as aerobic performance following blood transfusion (Buick et al. 1980; Calbet et al. 2006; Lundby and Robach 2015), it stands to reason that the increases in [Hb] resulting from splenic contraction in XC skiers could offer functional advantages. The dynamic regulatory capacity of the spleen in controlling circulating [Hb] becomes particularly interesting in the context of XC skiing performance; XC skiing is often performed on tracks characterized by a fluctuation between uphill and downhill segments throughout the race.

While previous research (Stewart et al. 2003) observed a 30% splenic contraction at 60%-$\dot{V}$O_2max_, and a 56% splenic contraction resulted following maximal intensity cycling, indicating varying degrees of contraction depending on exercise level, the potential dynamic splenic function has never been studied relating to XC skiing. We recently investigated spleen volume and contraction and [Hb] response during apneas and maximal exercise in elite biathletes, observing a greater exercise-induced splenic contraction compared with apnea (Holmström et al. 2021). Although we found that the biathletes had significantly larger spleens (214 mL) compared with a non-trained control group (157 mL) and also greater splenic contraction than that of controls, we were unable to indicate any positive effects on their skiing performance. However, any positive effects on biathlete´s performance could be unrelated to skiing; in biathlon, there is a component of apnea involved in the shooting events, which may benefit from enhanced splenic function, and possibly cause a training effect similar to that found following regular apnea training (Bouten et al. 2019).

Spleen volume and function have never previously been studied in XC skiers, and the precise relationship between splenic contraction and the associated [Hb] increase, relative to exercise intensities, remains unexplored. Therefore, we aimed to investigate splenic contraction and the concomitant [Hb] increase in well-trained XC skiers during four exercise intensities and during apnea.

## Materials and methods

### Participant recruitment and ethics

Fourteen male, well-trained XC skiers volunteered to participate in this study (mean (SD); age: 25 (4) years, height: 183 (7) cm, and body mass: 77 (6) kg). The participants were recruited by personal communication with competing or former competing well-trained cross-country skiers. The inclusion criteria were 18–40 years old with $\dot{V}$O_2max_ > 60 ml·kg^−1^·min^−1^ and being experienced in classic diagonal treadmill roller-skiing. Exclusion criteria were splenectomy or any known disease or illness. Calculations of statistical power on the main variables were made a priori, after which decision was made to recruit a maximum of 15 participants to obtain a minimum of 12 participants. After responding to the first contact and agreeing to give out their email addresses, an information sheet for participants was distributed, and on arrival to the laboratory, an informed consent form was signed. This study was conducted in accordance with the Declaration of Helsinki and ethical approval was received through the regional review board in Umeå, Sweden (dnr 2020-00044).

### Study design

The current study design included three parts divided over 2 days. The first day (test day 1) consisted of an apnea test and a $\dot{V}$O_2max_ test. The second day (test day 2), 2 to 10 days later, consisted of three submaximal intensity levels on a roller-skiing treadmill. The order of the intensity levels was randomized, and individual levels were calculated to correspond to 55, 70, and 85% of the participants’ measured $\dot{V}$O_2max_.

### Procedures and measurements

On arrival at the laboratory, all participants were informed about the procedure and they signed the informed consent, after which measurements of body mass on a precision scale (Seca 764, Hamburg, Germany) and height were collected with a stadiometer attached to a wall. They were then equipped with a peripheral venous catheter, typically in the basilic or cephalic vein in the right arm fold. Seated down on a cushioned chair, they started the first resting period and filled out the health form, confirming that they felt completely healthy at the time. After 20 min of sitting rest, the first baseline splenic measurements and blood samples were collected.

#### Blood sampling

The venous blood samples were drawn with a 5-ml syringe, after which it was manually transferred into test tubes and cuvettes. A saline solution was used to flush the catheter and a waste sample of 1 ml was drawn from the arm before each sample. At each sampling occasion, a sample of 4 ml blood was drawn and transferred into three kinds of test tubes. First, a 500-µl sample was taken for analysis in a Horriba Micros 60 hematology analyzer (Diamond Diagnostic, Holliston, MA, US). Second, a blood lactate (Hla) sample (20 µl) was taken and measured with a Biosen S-line (EKF diagnostic GmbH, Magdeburg, Germany), which was calibrated with a standard solution of Hla (12 mmol·L^−1^) prior to each analysis. Third, the remainder of the sample (~ 3.5 ml) was transferred into a 3.5-ml vacutainer with a coagulant solution and analyzed for serum albumin (S-albumin) and total protein content (S-protein) with a Cobas Pro equipment (Roche Diagnostics, Indianapolis, IN, US). Serum S-protein and S-albumin concentration levels were analyzed to identify the hemoconcentration changes due to possible plasma volume changes.

#### Splenic measurements

**Fig. 1:**
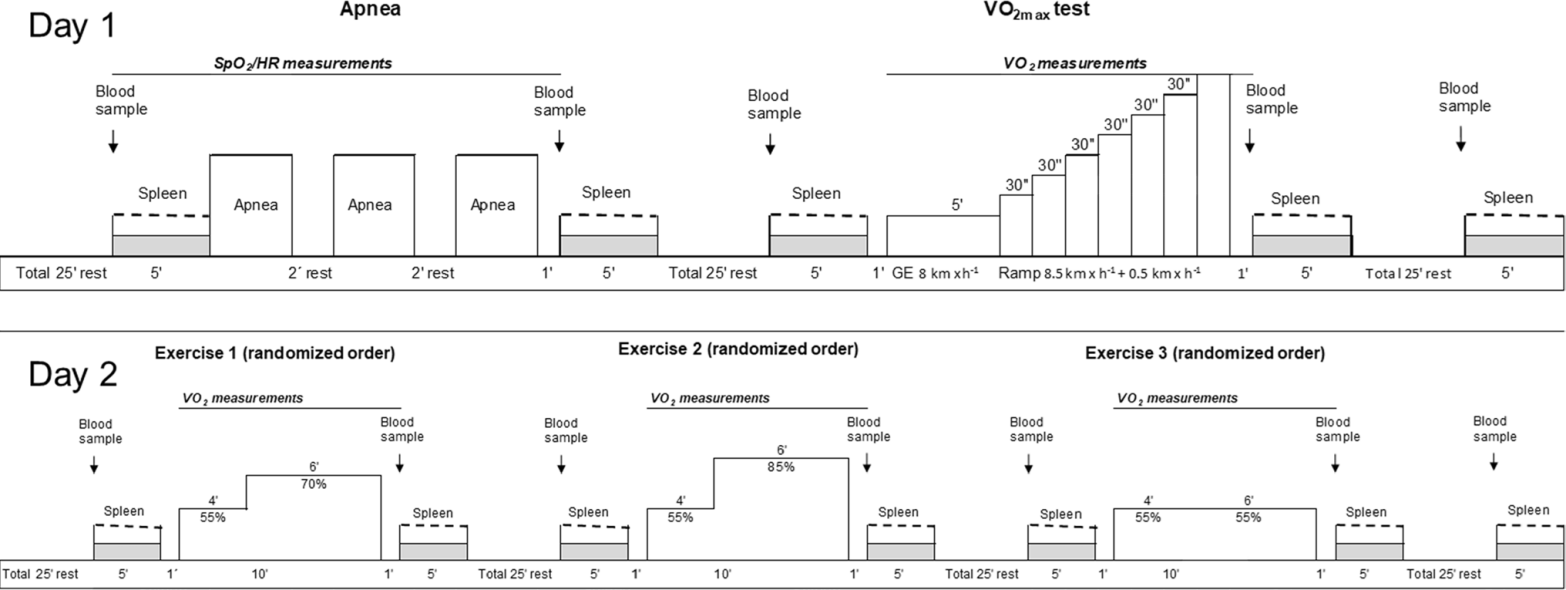
Protocol overview of test day 1 and day 2. Arrows showing time points where blood samples were taken (for [Hb], serum albumin, and serum whole protein). Dashed lines show ultrasound measurements of the spleen; filled lines show measurements of ${\dot{\text{V}}}$O_2_, SpO_2_, and HR; numbers show number of minutes (‘) or seconds (‘’) of rest and conditions. Day 1 was always performed in the same order, whereas exercise conditions on day 2 were randomized in order. EX 55, EX 70, and EX85 = Exercise intensity corresponding to 55, 70, and 85% of ${\dot{\text{V}}}$O_2max_

Spleen volume was measured from the dorsal side via ultrasonic imaging (M-Turbo Ultrasound system, FUJIFILM SonoSite Inc., Bothell, WA) with the probe: C60x/5–2 MHz (SonoSite Inc., Bothell, WA, USA), by an experienced sonographer (PH). Two ultrasonic images were recorded each minute for determination of the following maximal three-axial diameters: length (L), width (W), and thickness (T). The baseline (resting) spleen volume was measured each minute during the last 5 min of the 20 min of rest before each test and was used as an average to compare with values obtained after apnea and exercise (Fig. 1). Spleen measurements were collected continuously for 5 min immediately following apnea and starting at 1 min following exercise. Spleen volume measurements were also made continuously between 20 and 25 min following the exercise bouts to obtain splenic recovery measurements (Fig. 1.).

### Cardiorespiratory measurements

Throughout the apnea test, peripheral oxygen saturation (S_p_O_2_) and heart rate (HR) were continuously measured using a combined pulse oximeter (Medair LifeSense LS1-9R, Medair AB, Delsbo, Sweden) attached to the participant’s finger. Measurements of respiratory variables were made using an AMIS 2001 model C (Innovision A/S, Odense, Denmark) metabolic cart. Calibration of the metabolic carts flowmeter was performed with a 3-L syringe (Hans Rudolph, Kansas City, Missouri, USA) over a range from low to high flow rates and the gas analyzers were calibrated before each test with a gas mixture of 16.0% oxygen and 4.5% CO_2_ in N_2_ (Strandmöllen, Ljungby, Sweden). The ambient conditions were monitored before each test with an external device (Vaisala PTU 200, Vaisala Oy, Helsinki, Finland), and corrections were made to the internal ambient calibration of the metabolic cart. HR was monitored with a Polar V800 chest strap apparatus (Polar Electro Oy, Kempele, Finland). The resting periods were made sitting on a cushioned chair where all blood samples and spleen volume measurements were collected.

### Protocol

#### Apnea test

The apnea test consisted of three maximal voluntary static and dry apneas with 2 min of rest in between, which were performed in a sitting position following 25 min of sitting rest (Fig. 1). The participants were instructed to exhale completely, inhale one deep but not maximal breath and then to hold the breath as long as they could before voluntary termination (Schagatay et al. 2005). Through a pulse oximeter attached to the participant’s finger, a research assistant monitored the HR and S_p_O_2_. If the S_p_O_2_ fell below 60% at any point, the participants would be told to terminate the breath-hold and resume breathing. This never occurred. To avoid psychological effects, no feedback concerning apnea duration was provided to the participants.

#### $\dot{V}$*O*_*2max*_* test*

The roller skiing tests were all performed on a motor-driven roller skiing treadmill (Rodby Innovation AB, Vänge, Sweden), and Pro-Ski C2 (Sterners, Dala-Järna, Sweden) roller skis were equipped with Prolink (Salomon, Annecy, France) bindings. The participants wore a safety harness, during all tests, which was connected by a rope to an emergency brake. Following an additional 25 min of seated rest and sampling (Fig. 1.), a combined GE and $\dot{V}$O_2max_ test protocol was performed to establish the maximal aerobic capacity as well as for calculating roller skiing speeds for test day 2. The two-phased automated protocol started with a 5-minute constant speed GE measurement period and ended with a $\dot{V}$O_2max_ ramp time to exhaustion (TTE) test. The GE section was performed at 8 km·h^−1^ and after 5 min, the ramp part started and increased 0.5 km·h^−1^ each 30 s. All roller skiing tests were performed at a constant incline of seven degrees to ensure basis for a good diagonal skiing technique (Andersson et al. 2017). The test was terminated by an experienced test leader (HL) when the participant no longer could keep up with the speed of the treadmill.

#### Exercise intensities

To determine the effects of exercise intensity, three different submaximal exercise levels were performed in a randomized order during test day 2 (Fig. 1.). The participants commenced the test day as described in day 1, after which three exercise levels were performed. Roller skiing speeds were calculated according to a model based on the work by Andersson et al. (2017), to correspond to 55% (EX55), 70% (EX70), and 85% (EX85) of each participant’s individual $\dot{V}$O_2max_ from test day 1. All submaximal levels started with 4 min of warm-up at 55% intensity followed by 6 min at a randomized order. The low intensity was chosen to avoid the risk of an added sympathetic splenic response caused by starting at high intensity. Seated rest for 25 min followed each level of exercise. Blood samples were taken at 5 min before and 1 min after exercise, and spleen measurements were taken for 5 min prior to exercise as well as for 5 min starting 1 min after exercise. One minute was needed for the participant to get to the seated position after being assisted in taking off roller skis, poles, safety harness, and the mouthpiece from the metabolic cart.

### Analysis

The splenic diameter measurements obtained were used for volume calculations via the Pilström equation as follows:

$V$_spleen_ = Lπ(WT-T^2^)/3 (Schagatay et al. 2005).

The splenic measurements were analyzed either as an average of five samples (baseline) or simply as the first spleen size following apnea and exercise. This method has recently been observed to have high reliability between repeated measurements (Holmström et al. 2022). Each condition is associated with a preceding baseline resting period, wherein splenic and hematological baseline values refer to measurements during this rest period prior to each condition separately. Individual splenic contraction was then calculated as an absolute (mL) and relative (%) change in the volume after the apnea and exercise from the associated baseline volume. Spleen volume was expressed in mL and in relation to body height (mL·cm^−1^).

[Hb], Hla, S-albumin, and S-protein samples were analyzed as one measurement within the recommended time for each sample type. $\dot{V}$O_2max_ was calculated as the average of the highest consecutive 30 s during the $\dot{V}$O_2max_ protocol. The criterion for reaching $\dot{V}$O_2max_ was a respiratory quotient (RQ) > 1.1, and Hla > 8 mmol·L^−1^. GE was calculated using treadmill speed and inclination, rolling resistance of roller skis, and metabolic data of $\dot{V}$O_2_ and RER (Andersson et al. 2017).

### Statistical analysis

Statistical analyses were performed using IBM SPSS Statistics (Version 27.0; IBM Corporation, NY, US) with a significance level set to *α* < 0.05. Data were reported as mean (SD) and normal distribution of data was assessed using the Shapiro–Wilk test (*p* < 0.05). Repeated-measures two-way ANOVAs (within factors: condition; time) were used to identify if conditions (apnea; exercise intensities) influenced splenic contraction and [Hb]. Significant interactions were followed up with simple main effect analyses with one-way repeated-measures ANOVAs which were adjusted for multiple comparisons with Bonferroni adjustment. The magnitude of the effects was estimated with Cohen’s d effect size (ES), computed as the mean difference divided by the pooled SD. ESs are presented with 95% confidence intervals (CIs), and an ES of 0.2–0.3 was considered small, 0.4–0.7 as medium, and 0.8 as large (Lee 2016). Pearson’s product-moment correlation coefficients (r) were used to quantify associations between splenic contraction and [Hb] with XC skiing performance indicators ($\dot{V}$ O_2max_).

## Results

Spleen volume, [Hb], S-albumin, and S-protein changes are presented in Table 1. There was an interaction effect between conditions and time on splenic contraction (*F*_(5.09,66)_ = 9.497, *p* < 0.001) and [Hb] (*F*_(3.45,45)_ = 12.169, *p* < 0.001), showing that splenic contraction and [Hb] changes are influenced by exercise intensity and apnea.

**Table 1:** Differences within and between conditions

	*n*	Mean (SD)	Δ Mean (SD)	Δ sign	95% CI	*p*-value	ES	95% CI
Baseline								
Spleen volume (ml)	14	266 (35)						
Hb (g·L^−1^)	14	140 (7)						
S-albumin (g·L^−1^)	14	41 (2.3)						
S-protein (g·L^−1^)	14	67 (4.4)						
Apnea (A)								
Spleen volume (ml)	14	211 (8)	− 55 (5)	BC	− 38 to − 72	< 0.001	− 1.5	− 2.3 to − 0.7
Hb (g·L^−1^)	14	146 (1.5)	4.6 (0.8)	BCD	2.4 to 6.9	< 0.001	0.8	0.0 to 1.6
S-albumin (g·L^−1^)	14	41 (2.3)	1.4 (0.4)	BC	0.4 to 2.5	0.006	0.6	− 0.1 to 1.4
S-protein (g·L^−1^)	13	70.7 (4.1)	1.9 (0.5)		0.5 to 3.2	0.008	0.3	0.5 to 3.2
${\dot{\text{V}}}$O2max (B)								
Spleen volume (ml)	14	167 (25)	− 94 (7)	ACDE	− 73 to − 115	< 0.001	− 2.8	− 3.8 to − 1.7
Hb (g·L^−1^)	14	152 (1.5)	11 (0.8)	A	9.0 to 13.4	< 0.001	1.7	0.8 to 2.5
S-albumin (g·L^−1^)	14	45.6 (0.5)	4.5 (0.5)	A	3.1 to 6.0	< 0.001	1.8	0.9 to 2.6
S-protein (g·L^−1^)	14	76.3 (4.4)	8.3 (0.9)	A	5.9 to 10.7	< 0.001	1.6	0.7 to 2.4
Ex85 (C)								
Spleen volume (ml)	14	196 (25)	− 73 (4)	AB	− 61 to − 85	< 0.001	− 2.5	− 3.5 to − 1.5
Hb (g·L^−1^)	14	150 (2.0)	11.4 (0.6)	ADE	9.8 to 12.9	< 0.001	1.6	0.7 to 2.4
S-albumin (g·L^−1^)	14	44.8 (0.6)	3.8 (0.3)	AE	3.1 to 4.6	< 0.001	1.7	0.8 to 2.5
S-protein (g·L^−1^)	14	73.6 (5.0)	7.5 (0.5)	AE	6.0 to 9.0	< 0.001	1.7	0.8 to 2.5
Ex70 (D)								
Spleen volume (ml)	14	207 (29)	− 62 (4)	B	− 48 to − 75	< 0.001	− 2.0	− 2.9 to—1.1
Hb (g·L^−1^)	14	148 (2.2)	8.8 (0.7)	ACE	6.9 to 10.8	< 0.001	1.2	0.3 to 1.9
S-albumin (g·L^−1^)	14	43.8 (0.6)	2.9 (0.3)		2.1 to 3.7	< 0.001	1.2	0.4 to 2.0
S-protein (g·L^−1^)	14	72.4 (4.6)	5.4 (0.6)	A	3.6 to 7.1	< 0.001	1.3	0.4 to 2.0
Ex55 (E)								
Spleen volume (ml)	14	212 (10)	− 55 (6)	B	− 35 to − 74	< 0.001	− 1.6	− 2.3 to − 0.7
Hb (g·L^−1^)	14	146 (7.5)	6.7 (0.6)	BCD	5.0 to 8.3	< 0.001	0.9	0.1 to 1.7
S-albumin (g·L^−1^)	14	43.2 (2.3)	2.1 (0.3)	BC	1.3 to 3.0	< 0.001	1.0	0.2 to 1.7
S-protein (g·L^−1^)	14	70.8 (4.1)	3.8 (0.4)	BC	2.7 to 4.9	< 0.001	− 0.2	− 0.9 to 0.6

ΔMean (SD) = Difference between the measurement 1 min post each condition and the associated baseline value. A = indicate *p* < 0.05 difference compared to apnea. B = indicate *p* < 0.05 difference compared to V̇O_2max_; C = indicate *p* < 0.05 difference compared to EX85; D = indicate *p* < 0.05 difference compared to EX70; E = indicate *p* < 0.05 difference compared to EX55

### Spleen volume and [Hb] changes within conditions

**Fig. 2:**
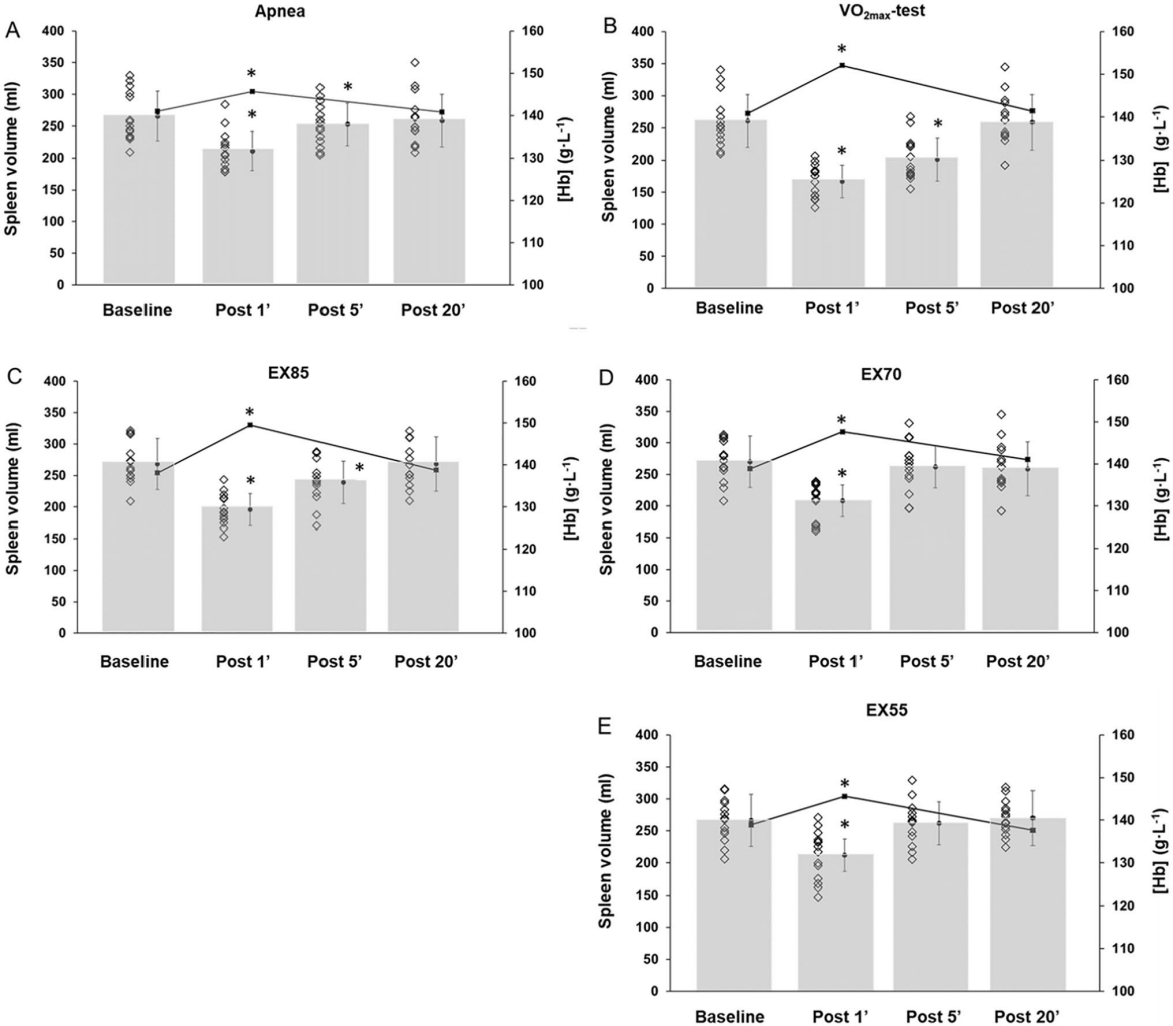
Spleen volume and [Hb] changes from baseline to recovery. A = Change after apnea; B = Change after ${\dot{\text{V}}}$O_2max_ test; C = Change after 85% intensity; D = Change after 70% intensity; E = Change after 55% intensity. Unfilled diamond = Individual spleen volume. Filled circle = Mean (SD) spleen volume. Filled square with line = Mean [Hb] change. Baseline > 1 min prior to conditions. Post 1, Post 5, Post 20 = 1, 5, and 20 min post conditions. * indicates significant change (*p* < 0.05) compared to baseline

All conditions induced significant splenic contractions and transient [Hb] elevations (Table 1). Baseline spleen volume was similar between all conditions at 266 ± 35 ml. Spleen volume was reduced by 20.4% following three maximal apnea and had not returned to baseline values following 5 min of rest (*p* = 0.028, Fig. 2A). During apneas, [Hb] increased by 3.3% (*p* < 0.001). Following maximal exercise ($\dot{V}$O_2max_), spleen volume decreased by 35.8% (*p* < 0.001) and had not returned to baseline values following 5 min of rest (Fig. 2B). During maximal exercise, [Hb] increased by 8.1% (*p* < 0.001, Fig. 2B). Following EX85, spleen volume decreased by 27.1% and was still reduced following 5 min of rest (*p* < 0.001, Fig. 2C). [Hb] increased by 8.3% (*p* < 0.001, Fig. 2C). During EX70, spleen volume was reduced by 23.0% (*p* < 0.001) and returned to baseline following 5 min of rest (Fig. 2D). During EX70, [Hb] increased by 6.4% (*p* < 0.001, Fig. 2D). Finally, the spleen was reduced by 20.7% in size following EX55 (*p* < 0.001), after which it returned to baseline values within 5 min of rest (Fig. 2E). The [Hb] increase at EX55 was 4.8% (*p* < 0.001, Fig. 2E).

### Splenic and [Hb] responses between conditions

**Fig. 3:**
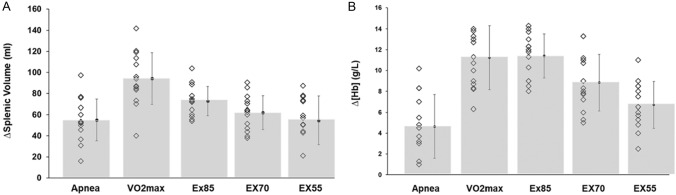
Individual change in spleen volume and [Hb] for each condition. A = magnitude of splenic contraction; B = [Hb] elevation. Filled dots indicate mean (SD)

The $\dot{V}$O_2max_ test resulted in a greater splenic contraction compared with all other conditions (apnea, 95% CI [12.3–66.3], *p* = 0.003; EX85, 95% CI [1.1–41.5], *p* = 0.035; EX70, 95% CI [12.8–51.6], *p* < 0.001; EX55, 95% CI [14.3–65.0], *p* = 0.002). Splenic contraction during EX85 was higher than that of apnea (95% CI [0.48–35.4], *p* = 0.042). The apnea-induced splenic contraction displayed a similar magnitude as EX55 and was lower compared with values during the $\dot{V}$O_2max_ test and EX85 (Fig. 3A).

The [Hb] increase following the $\dot{V}$O_2max_ test was higher compared with that of the EX55 (95% CI [1.4–7.6], *p* = 0.003) and apnea (95% CI [2.5–10.6], *p* = 0.001, Fig. 3B). While [Hb] elevations during EX85 was higher compared with EX70 (95% CI [0.1–5.0], *p* = 0.034), EX55 (95% CI [2.7–6.6], *p* < 0.001) and compared with values during the apnea test (95% CI [3.2–10.3], *p* < 0.001), it was similar to values following the $\dot{V}$O_2max_ (NS). [Hb] increases during the EX70 were also higher compared with that of EX55 (95% CI [0.4–3.9] (*p* = 0.01) and apnea (95% CI [0.8–7.6] (*p* = 0.01, Fig. 3B).

### S-albumin and S-protein responses

Baseline S-albumin and S-protein values were similar for each condition (Table 1, NS). Both S-albumin and S-protein increased following each condition (Table 1, *p* < 0.01), indicating plasma volume reductions. In addition, the magnitude of S-albumin and S-protein concentration increases varied across conditions (Table 1), where values following the $\dot{V}$O_2max_ test were significantly higher compared with values during the apneas (*p* = 0.013, *p* = 0.002) and EX55 (*p* = 0.033, *p* = 0.006) for S-albumin and S-protein respectively. S-albumin and S-protein responses had returned to baseline following 20 min of rest.

### Performance measures

The mean apneic duration was 85 (36) s, 103 (46) s, and 118 (50) s for the three apneas, respectively, and thereby increased across the series with 18 (5) s (95% CI [33–4 s] *p* = 0.012) from apnea one to two and 33 (8) s (95% CI [12–55], *p* = 0.003) from apnea 2 to 3. During the $\dot{V}$O_2max_ test, the mean GE was 20.8 (0.9)%, average TTE duration was 669 (24) s, and the absolute and relative $\dot{V}$O_2max_ were 5.34 (0.34) L·min^−1^ and 69 (3) ml·kg^−1^·min^−1^, respectively. The $\dot{V}$O2% was 56 (2)%, 72 (2)%, and 88 (2)%, respectively, during EX55, EX70, and EX85. Accumulated Hla following the $\dot{V}$O_2max_ test was 10,1 (1,5) mmol ·L^−1^, while it was 3,3 (0,8) mmol ·L^−1^, 1,0 (0,2) mmol ·L^−1^, and 0,7 (0,2) mmol ·L^−1^ for EX85, EX70, and EX55, respectively.

### Correlational analysis

Baseline spleen volume was positively associated with apnea-induced splenic contraction (*r* = 0.552, *p* = 0.041) and maximal exercise-induced contraction ($\dot{V}$O_2max_-test, *r* = 0.706, *p* = 0.005), while no association existed between spleen volume and submaximal exercise-induced contractions. $\dot{V}$O_2max_ was positively associated with TTE duration (*r* = 0.763 *p* = 0.001) and [Hb] increase at EX85 (*r* = 0.533, *p* = 0.049), EX70 (*r* = 614, *p* = 0.019), and EX55 (*r* = 0.630, *p* = 0.016). Furthermore, the [Hb] increase was positively associated with increases in S-albumin and S-protein concentration, respectively, during both the apnea test (*r* = 0.615, *p* = 0.019; *r* = 0.710, *p* = 0.007) and the $\dot{V}$O_2max_ test (*r* = 0.876, *p* < 0.001; *r* = 0.746, *p* = 0.002).

## Discussion

The primary findings of this study were that: (1) there are differences in the magnitudes of splenic contraction and [Hb] increases among the exercise intensities, with progressively greater responses at higher intensities and significantly greater responses at EX85 ([Hb]) and $\dot{V}$O_2max_ (spleen) compared with the lower intensities; (2) the highest exercise intensities, $\dot{V}$O_2max_ and EX85, also resulted in greater splenic contraction and [Hb] increases compared with apnea.

### Baseline spleen volume

We observed baseline (resting) spleen size of 266 mL in the XC skiers. This is larger than what we observed previously in elite biathletes (214 mL) compared to untrained controls (157 mL; (Holmström et al. 2021). The latter investigation also showed that elite biathlete´s spleen size was positively associated with their annual endurance training volume, which was not present in the non-athletes. Accordingly, we suggested that enhanced spleen size in XC skiers could be a long-term effect in response to their high volume of endurance exercise (Holmström et al. 2021). However, compared with high-altitude climbers (367 ml) (Schagatay et al. 2020) and freedivers (336 ml) (Schagatay et al. 2012), the spleens of our skiers and the biathletes are relatively smaller. Exercise may thus have a long-term effect on spleen volume, while still not inducing similar magnitudes as the exposure to regular hypoxia during high-altitude climbing or freediving.

This variability in spleen volume across groups exposed to different environments and level of stress raises the question whether similar variations in spleen size and particular contraction exist within endurance athletes performing at different exercise intensities. It appears conceivable that the magnitude of the splenic contraction is dependent on the exercise intensity, i.e., when oxygen demand increases the magnitude of the splenic contraction is increased with concomitant increase in [Hb], to meet the higher oxygen demand. This line of thinking implies that splenic contraction is of practical importance during endurance performance. While it seems logical that transient Hb increases during bouts of exercise could be beneficial for the endurance athlete, several studies report no effects on endurance performance following apnea (Sperlich et al. 2015; Bouten et al. 2019). It is likely that the effects of apnea could possibly be more evident on recovery mechanisms than peak performance.

### Exercise-induced splenic contraction and [Hb] increase

The notion that splenic contraction confers a functional advantage during endurance exercise gains credibility when considering the physiological connection between aerobic performance and increased oxygen-carrying capacity via higher [Hb], as demonstrated previously (Buick et al. 1980; Calbet et al. 2006; Lundby and Robach 2015). Furthermore, an earlier study noted a decrease in RBCs within the spleen concurrently with a rise in hematocrit as exercise intensity increased (Laub et al. 1993), suggesting that splenic contraction contributes to the elevation of [Hb] during endurance exercise. This discovery was substantiated by our own study, which found a strong association between spleen size and the magnitude of [Hb] increase during maximal exercise in elite biathletes (Holmström et al. 2021), indicating that a larger spleen exerts a more prominent influence on exercise-induced [Hb] compared to a smaller one. When coupled with the observation of increased spleen size in elite endurance athletes, which indeed decreases during exercise (Holmström et al. 2021), it becomes plausible that splenic contraction may have a functional advantage during endurance exercise performance.

We found that the 35% splenic contraction during the $\mathop {\text{V}}\limits^{.}$O_2max_ test was greater compared with all other exercise intensities, and also compared to apnea in the XC skiers, a stimulus often used to trigger spleen contraction. While no significant differences could be distinguished between the lower exercise intensities, a clear trend was evident, indicating a progressively increased splenic contraction from the lowest to maximal intensity. Our observed splenic contractions match well with the 34% we observed previously in elite biathletes (Holmström et al. 2021), although smaller than the 56% observed by Stewart et al. (2003) and around 50% observed by Jahic et al. (2019) in runners. Stewart et al. (2003) also observed a splenic contraction of around 30% on an intensity corresponding to 60%${\dot{\text{V}}}$O_2max_, whereas Jahic et al. (2019) observed a 20–40% reduction at the aerobic threshold. We observed a 20% splenic contraction already at an intensity corresponding to 55%${\dot{\text{V}}}$O_2max_, but never more than 27% during the submaximal exercise intensities, which may be a result of small intensity increments and a relatively small sample size. All exercise intensities resulted in significant splenic contractions with high effect sizes in our study, while we did not observe significant differences. These differences suggest that there is a dose-dependent relationship across a variety of exercise intensities, in accord with earlier findings (Stewart et al. 2003).

The greater magnitude of splenic contraction at maximal exercise could possibly be a consequence of sympathetic regulation of the spleen (Stewart and McKenzie 2002). Nevertheless, the high Hla level observed during the $\dot{V}$O_2max_ test compared to the submaximal intensities could also indicate a splenic contraction due to a higher anaerobic contribution, indicating an oxygen deficiency creating greater demands of circulating [Hb]. However, this would also indicate that [Hb] would be higher at $\dot{V}$O_2max_ test compared with EX85, which was not the case in the present study. Holmström et al. (2021) showed a [Hb] increase by 11.2% following short-term maximal exercise in biathletes, which was higher than what studies previously have shown (Laub et al. 1993; Wolski 1999; Stewart et al. 2003). The present study showed no difference in [Hb] between maximal exercise (8.1%) and exercise around anaerobic threshold at EX85 (8.3%), which contradicts the theories of hemoconcentration due to an increased oxygen demand at maximal exercise. It could be speculated that the [Hb] response acts at lower intensities in well-trained athletes to increase oxygen transport already before the anaerobic threshold has been reached.

In addition, $\dot{V}$O_2max_ was strongly correlated with performance of the TTE test, agreeing with previous research, reporting that high-level XC skiers typically have high $\mathop {\text{V}}\limits^{.}$O_2max_ values (Sandbakk and Holmberg 2014; Tønnessen et al. 2015), underscoring their aerobic performances. There was a moderate correlation between $\dot{V}$O_2max_ and [Hb] increase at EX85, EX70, and even EX55, indicating that splenic-derived [Hb] elevations enhance the oxygen transportation in the blood, which in turn could be inducing the greater aerobic capacity on higher intensities. However, this correlation was not observed with [Hb] during the $\dot{V}$O_2max_ test.

### S-albumin and S-protein changes

S-albumin and S-protein measurements were collected to explore potential markers for exercise intensity-based reductions in plasma volume, consequently leading to an increase in [Hb]. Interestingly, both S-albumin and S-protein concentrations exhibited a similar trend to that of [Hb], showing a greater increase relative to [Hb] across all exercise intensities. An exception to this pattern was observed during apnea, where [Hb] increased more than S-albumin and S-protein. This observation prompts a compelling question: To what extent do these changes in [Hb] during exercise result from a reduction in blood plasma volume, and how much is attributed to splenic contraction?

Wolski (1999) postulated that 65–80% of the increase in hematocrit is a consequence of hemoconcentration due to reduced plasma volume, with the remainder arising from an augmentation in the circulating volume of RBC. Previous research has indicated significantly higher elevations in [Hb] during apnea and exercise in individuals with spleens compared to those who have undergone splenectomy (Agostoni et al. 1999; Schagatay et al. 2001; Baković et al. 2005), underscoring the importance of the splenic response in enhancing [Hb]. However, in the current study, the change in [Hb] exhibited a correlation with changes in S-Albumin and S-Protein concentrations, rather than with splenic contraction. This suggests that a substantial portion of the increased [Hb] during skiing exercise may be attributed to hemoconcentration resulting from rapid reductions in plasma volume. Nonetheless, the transient increase in [Hb], which returns to baseline within minutes after exercise or apnea, provides evidence that splenic contraction plays a role in elevating [Hb] during exercise.

### Methodological considerations

The variability among individuals in resting spleen size, as evidenced by our study, where baseline spleen size ranged from 208 to 350 ml, signifies substantial individual differences, which make it necessary to investigate a large sample to generalize effects. There is a small pulsatile variation in spleen size also during rest, but all baseline spleen measurements were obtained as an average over 5 consecutive minutes, a practice aimed at minimizing the potential impact of pulsatile changes and possible measurement errors. All data were collected by the same sonographer, which further reduced measurement errors. The current data collection procedure has recently demonstrated high reliability in repeated tests (Holmström et al. 2022).

The absence of a significant correlation between splenic contraction and [Hb] increase may well be a result of a small sample size. In research involving well-trained endurance athletes, recruiting a large sample can be particularly challenging. Furthermore, the effect of exercise and voluntary apnea on [Hb] is generally modest, often in the range of approximately 2% to 8%, and an outlier with an extreme value could exert a substantial influence on the overall results.

When comparing the data derived from the apnea test and the $\mathop {\text{V}}\limits^{.}$O_2max_ test, it is important to acknowledge that these assessments took place on the same day (day 1), with the apnea test conducted before the $\mathop {\text{V}}\limits^{.}$O_2max_ test for all participants. Although both spleen size and all hematological variables returned to their baseline levels within 20 min of rest, it is worth noting that they were not randomized. As a result, there may be a slightly different splenic response due to a potential carry-over effect from the apnea session, which could conceivably influence the outcomes of the ${\dot{\text{V}}}$O_2max_ test. However, considering that both baseline measurements and recovery measurements, taken after 20 min of rest, exhibited no significant differences, we believe any such influence to be minimal.

## Conclusion

Our findings reveal a discernible link between the extent of splenic contraction and the elevation in [Hb], which varies in response to exercise intensity that can effectively be categorized into two distinct domains: (1) submaximal intensity exercise, including apnea, and (2) maximal effort exercise. This observation underscores the presence of a dose-dependent connection between splenic contraction and the concurrent increases in [Hb] and exercise intensity. However, it is noteworthy that the changes in [Hb] were not solely attributable to changes in spleen volume. This suggests the involvement of other potential sources contributing to the elevation of circulating [Hb]. To gain a more comprehensive understanding, future research should investigate the extent to which the [Hb] increase results from splenic contraction and how exercise-induced changes in plasma volume might influence [Hb].

## Perspectives

Our findings may hold practical significance, in the context of endurance performance in general and for XC skiing in particular. XC skiing is distinctive due to its inherent variations in exercise intensity and subsequent aerobic and anaerobic contributions (Andersson et al. 2017), a result of the undulating ski tracks that feature uphill and downhill segments. These fluctuations in exercise intensity correspond with changes in metabolic demand, whereby high-intensity uphill segments benefit from splenic contraction, enhancing oxygen-carrying capacity through elevated [Hb]. While during downhill segments with lower metabolic demand, the spleen may instead relax and store circulating RBC, reducing blood viscosity. In addition, the splenic contraction could serve as a valuable warm-up strategy. A pre-exercise apnea series, could boost circulating [Hb], improving oxygen-carrying capacity before the exercise begins. This perspective underscores the dynamic role of the spleen in regulating circulating [Hb] in endurance exercise and XC skiing performance. While the precise functional advantage of the spleen in endurance exercise remains unclear, previous observations of improved ${\dot{\text{V}}}$O_2_ kinetics during short-duration exercise following apnea (Bouten et al. 2019) and enhanced spleen size and greater contraction in elite skiers (Holmström et al. 2021) support the practical implications of splenic function described above.

## Data Availability

Data supporting this study are available upon request to the authors.

## Abbreviations

ANOVA: Analysis of variance
ES: Effect size
EX55: 55% Exercise intensity
EX70: 70% Exercise intensity
EX85: 85% Exercise intensity
GE: Gross efficiency
Hb: Hemoglobin
[Hb]: Hemoglobin concentration
Hla: Blood lactate
HR: Heart rate
NS: Non-significant
RBC: Red blood cell
RER: Respiratory exchange ratio
RQ: Respiratory quotient
SpO2: Peripheral oxygen saturation
S-protein: Serum protein
S-Albumin: Serum albumin
TTE: Time to exhaustion
$$\dot{V}$$O2: Oxygen uptake
$\dot{V}$O2max: Maximal rate of oxygen uptake
XC: Cross-country
